# Late Engagement of CD86 after Influenza Virus Clearance Promotes Recovery in a FoxP3^+^ Regulatory T Cell Dependent Manner

**DOI:** 10.1371/journal.ppat.1004315

**Published:** 2014-08-21

**Authors:** Emily K. Moser, Matthew M. Hufford, Thomas J. Braciale

**Affiliations:** 1 The Beirne B. Carter Center for Immunology Research, The University of Virginia, Charlottesville, Virginia, United States of America; 2 Department of Pharmacology, The University of Virginia, Charlottesville, Virginia, United States of America; 3 Department of Microbiology, The University of Virginia, Charlottesville, Virginia, United States of America; 4 Department of Pathology, The University of Virginia, Charlottesville, Virginia, United States of America; Johns Hopkins University - Bloomberg School of Public Health, United States of America

## Abstract

Influenza A virus (IAV) infection in the respiratory tract triggers robust innate and adaptive immune responses, resulting in both virus clearance and lung inflammation and injury. After virus clearance, resolution of ongoing inflammation and tissue repair occur during a distinct recovery period. B7 family co-stimulatory molecules such as CD80 and CD86 have important roles in modulating T cell activity during the initiation and effector stages of the host response to IAV infection, but their potential role during recovery and resolution of inflammation is unknown. We found that antibody-mediated CD86 blockade in vivo after virus clearance led to a delay in recovery, characterized by increased numbers of lung neutrophils and inflammatory cytokines in airways and lung interstitium, but no change in conventional IAV-specific T cell responses. However, CD86 blockade led to decreased numbers of FoxP3^+^ regulatory T cells (Tregs), and adoptive transfer of Tregs into αCD86 treated mice rescued the effect of the blockade, supporting a role for Tregs in promoting recovery after virus clearance. Specific depletion of Tregs late after infection mimicked the CD86 blockade phenotype, confirming a role for Tregs during recovery after virus clearance. Furthermore, we identified neutrophils as a target of Treg suppression since neutrophil depletion in Treg-depleted mice reduced excess inflammatory cytokines in the airways. These results demonstrate that Tregs, in a CD86 dependent mechanism, contribute to the resolution of disease after IAV infection, in part by suppressing neutrophil-driven cytokine release into the airways.

## Author Summary

Influenza A virus (IAV) infection can cause severe inflammation and injury in the respiratory tract, which must be resolved and repaired for the host to fully recover after virus clearance. Evidence is emerging that host immune responses may regulate tissue repair and resolution of inflammation after IAV infection. Early in IAV infection, the co-stimulatory molecules CD80 and CD86 promote inflammation through triggering IAV-specific T cell responses, but no role for CD80/86 in recovery after virus clearance has been previously established. By in vivo antibody-mediated blockade of CD80 or CD86 after virus clearance, we found that engagement of CD86 (but not CD80) was required for optimal recovery after influenza infection. Furthermore, we determined that CD86 was essential for maintaining the FoxP3^+^regulatory T cell (Treg) population in the respiratory tract, and CD86-dependent Tregs promoted recovery by suppressing pulmonary inflammation and supporting regain of weight after virus clearance. In addition, we demonstrated that Tregs suppress neutrophils late after infection, preventing neutrophils from driving excess inflammatory cytokine release into the airways. Taken together, we propose a novel role for CD86 engagement late after IAV infection to promote resolution of inflammation and host recovery through a Treg-dependent mechanism.

## Introduction

Influenza A virus (IAV) infects and replicates in the respiratory tract, triggering a robust immune response. There is a large body of evidence to support the concept that fine control of the immune response to IAV is essential to prevent excessive tissue injury; while an immune mediated inflammatory response is required to effectively clear the virus, an exaggerated host response can result in bystander tissue damage and a subsequent decrease in lung function [1]–[3]. Therefore, many studies have attempted to determine whether inflammation can be controlled without compromising virus clearance [4]–[6]. Towards this goal, several components of the host immune system have been identified that enhance or reduce inflammation and injury; however, these studies have focused on the regulation of the initiation and/or effector phases of adaptive T cell responses to IAV. Thus, although significant inflammation and tissue damage are evident in the respiratory tract after infectious virus clearance, the factors involved in regulating the resolution of inflammation and injury after IAV clearance remain poorly characterized.

In a mouse model of IAV infection, recovery following virus clearance involves both tissue repair and the resolution of inflammation [7]. A number of studies have highlighted key factors and cell types during this complex process. In large part, local tissue repair within the respiratory tract is mediated by respiratory epithelial stem cells and fibroblasts [7], but there is provocative evidence to suggest that the host immune response is an important regulator of this process. For example, innate lymphoid cells and natural killer cells have been demonstrated to promote epithelial proliferation after IAV infection through the production of the growth factor amphiregullin [8] and the pro-wound healing cytokine IL-22 [9], respectively. In contrast to tissue repair, the resolution of inflammation has been considered to be primarily a consequence of the reduced IAV-specific effector T cell activity and decreased numbers of effector T cells following elimination of viral antigen (i.e. removal of the stimulus for inflammation (antigen) leads to a gradual return to homeostasis) [7]. However, in other models of acute lung injury (ALI), there is increasing evidence to support the importance of pro-resolving molecules such as Resolvin D1 and TGFβ in regulating clearance of inflammatory cells from the respiratory tract [10], [11]. Understanding the factors that control resolution of lung disease after IAV clearance could help identify therapeutic targets to promote faster recovery from severe respiratory viral infections.

CD86 and CD80 are members of the B7 family serving as co-stimulatory molecules. These ligands interact almost exclusively with the receptors CTLA4 and CD28, which in turn, are primarily expressed on CD4^+^ and CD8^+^ T cells. The CD86 and CD80 co-stimulatory molecules have been shown to enhance tissue inflammation by augmenting the response of conventional T effector cells [12] as well as to suppress inflammation through their ability to augment/sustain regulatory T cell (Treg) activity [13]. In the influenza model, CD80 and CD86 have been analyzed primarily in their capacity to support the induction of pro-inflammatory T effector responses and the expression of pro-inflammatory mediators by the T effector cells. During induction of T cell responses within the draining lymph node, CD28 engagement on naïve T cells by CD80/86 on antigen presenting cells (APCs) is required for a robust IAV-specific T cell response and efficient virus clearance [14]–[16]. In contrast, disruption of CD80/86 signaling to receptors on T cells after effector T cell generation dramatically reduces IAV-specific T cell effector activity in the respiratory tract (e.g. cytokine production and proliferation) but does not impact virus clearance or morbidity of mice [17], [18]. However, the role of CD80/86 co-stimulation in the recovery phase after IAV infection has not been previously evaluated. Furthermore, it is known that CD28 is required for the development, homeostatic maintenance, and proliferative expansion of Tregs, and CTLA4 is required for both expansion and expression of Treg suppressive function [13]. Importantly, although the ligand function of CD86 and CD80 demonstrates significant overlap, these two co-stimulatory molecules have been demonstrated to perform distinct roles under certain conditions. For example, in the NOD mouse model, CD80 and CD86 differ in their capacity to stimulate effector versus regulatory T cells [19], [20].

Treg cells are a subset of CD4^+^ T cells that express FoxP3 and can suppress inflammation in a number of disease models [21], [22]. During IAV infection, Treg cells have been shown to suppress innate and adaptive immune responses during the induction of primary [23], [24] and memory responses [25]; however, little enhanced pathology was noted in Treg depleted mice during primary IAV infection. Only in a model of memory responses to IAV infection did the enhanced effector CD8^+^ T cell response, resulting from elimination of Tregs at the time of secondary IAV challenge, cause increased morbidity in mice. The role of Treg cells, though, has not been evaluated during resolution of disease after virus clearance. In models of ALI (e.g. LPS-induced lung injury), Treg cells do promote resolution of injury in the respiratory tract, in large part by limiting accumulation of innate immune cells in the lung [26] and promoting non-inflammatory clearance of apoptotic neutrophils [11], [27]. Because of their immune-regulatory capabilities, Tregs are an obvious candidate to play a pivotal role in the resolution phase of infection after clearance of IAV.

In the present study, we initially evaluated the potential role of B7-family molecules CD80 and CD86 during resolution of disease (i.e. after virus clearance) by in vivo administration of a blocking monoclonal antibody directed to CD80 or CD86at day 8 post infection (p.i.), a time point shortly after IAV clearance within the respiratory tract [17]. We found that while in vivo administration of blocking monoclonal antibody directed to CD80 did not affect recovery, CD86 blockade at this time point lead to a delay in recovery as measured by regain of weight following virus clearance. Within the respiratory tract, this delay was associated with an increased number of leukocytes, augmented inflammatory cytokines, and a dramatic loss of the Treg population, with no corresponding impact on effector T cell activity, and this loss of Treg numbers correlated with a reduction in Treg proliferation. Importantly, adoptive transfer of Tregs into αCD86 treated mice rescued the effect of the blockade while acute depletion of Tregs during recovery mimicked the late αCD86 blockade model, supporting a role for Tregs in promoting recovery after virus clearance. The role of CD86 co-stimulation and Treg function during recovery from influenza infection is discussed.

## Results

### CD86 co-stimulation is required for optimal recovery following IAV infection

Previous work by our and other laboratories has established a role for the co-stimulatory molecules CD80 and CD86 in regulating IAV-specific T cell effector activity in the influenza-infected respiratory tract [17], [18]. Hufford et al. showed that simultaneous in vivo blockade of CD80 and CD86 (by monoclonal antibody administration) after initiation of T cell responses but before virus clearance within the infected lungs (i.e. day 5–6 p.i.) led to a dramatic reduction in T cell-derived cytokines, without any significant impact on virus control or morbidity of the mice. After infectious virus clearance and elimination of most virus-infected cells (i.e. day 8–10 p.i.), inflammation in the respiratory tract was gradually resolved, as evidenced by the reduction of inflammatory cells and cytokines, as well as the contraction of antigen specific effector T cells. However, the role of these residual immune cells and co-stimulatory ligands, such as CD80/86, during recovery and resolution of lung inflammation remained ill-defined.

**Figure 1:**
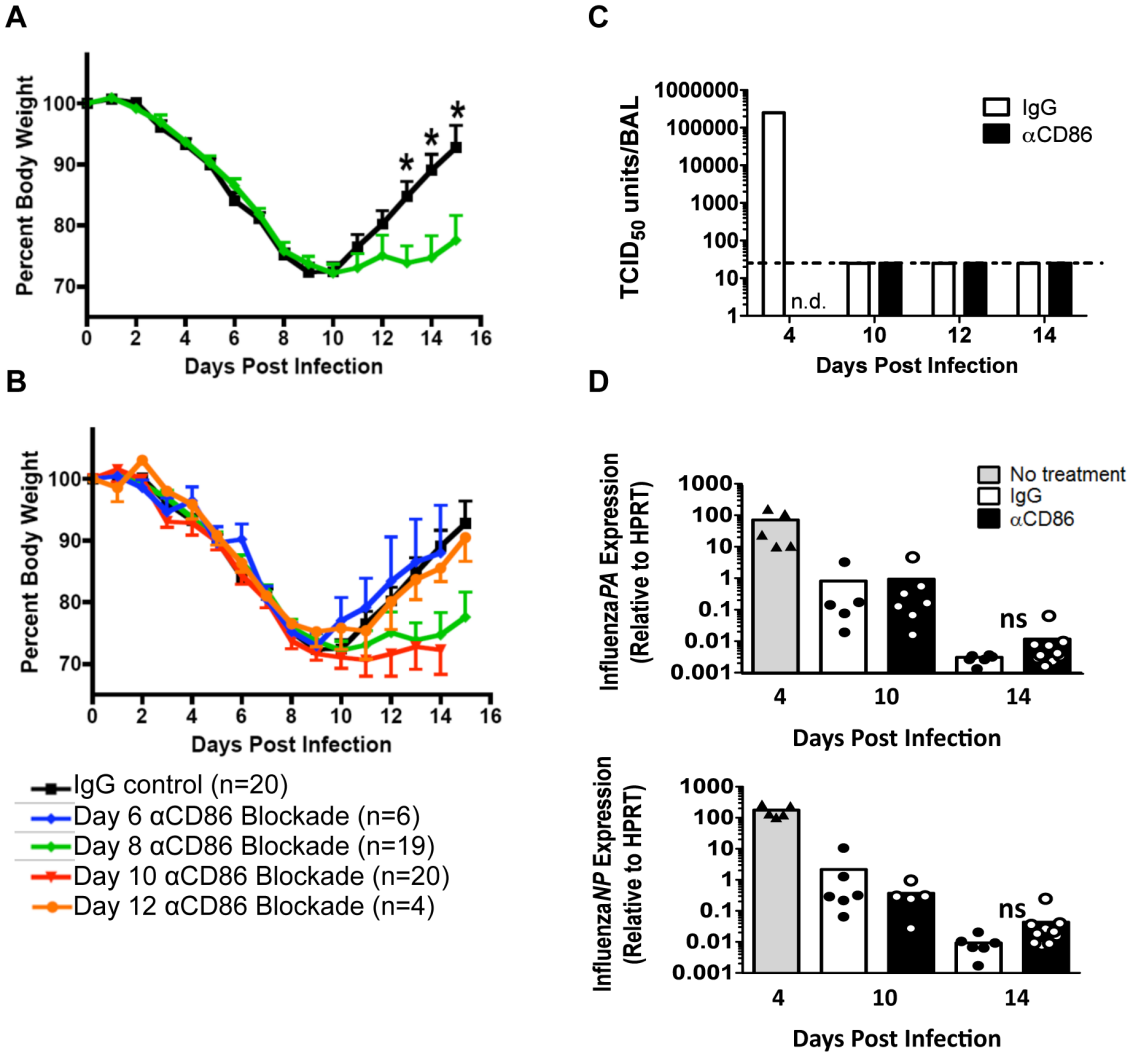
CD86 co-stimulation is required for optimal recovery following IAV infection. Balb/c mice were infected with 0.1 LD_50_ PR8 then treated with 200 µg αCD86 i.p. at various days p.i. (A–B) Weight loss of infected mice following CD86 blockade administered (A) on day 8 p.i. or (B) once on day 6, day 8 (same as in A, included as reference), day 10, or day 12 p.i(data are compiled from 2 or more independent experiments). (C–D) Mice were infected with 0.1 LD_50_ PR8 and treated with 200 µg αCD86 on day 9 p.i. At various days after antibody blockade, virus was quantified; (C) infectious virus in the BAL was measured by TCID_50_ assay (n = 2, n.d. = not done, dotted line indicates threshold of detection). (D) The influenza *PA* and *NP* genes were measured by qRT-PCR using whole lung homogenates (n = 5–10, compiled from 3 independent experiments, ns = not statistically significant). Samples from day 4 p.i. untreated mice were included as a positive control (n = 5, compiled from 2 independent experiments).

We wanted to determine what part, if any, co-stimulation (i.e. engagement of the CD80 and/or CD86 ligands) played in the regulation of inflammation during recovery following IAV clearance. To this end, we infected mice with a sublethal dose of the mouse adapted A/PR/8/34 (PR8) IAV strain, and we administered 200 µg αCD80 or αCD86 blocking antibodies intra-peritoneal (i.p.) at day 8 p.i., that is after virus clearance but before resolution of inflammation and recovery. CD80 blockade did not have any impact on recovery as reflected in weight gain during the recovery phase (data not shown). However, we found that CD86 blockade during the recovery phase led to prolonged morbidity as evidenced by a delay in weight gain (Figure 1A) although mice eventually regained 90–95% of their starting body weight by day 25 p.i. (data not shown). Importantly, this effect of in vivo CD86 blockade on morbidity was only evident during a narrow time frame following virus clearance and was not seen either when the blocking antibody was administered during active virus clearance from the lungs (day 6 p.i.) or when it was given late in the recovery phase (day 12 p.i.) (Figure 1B). Although CD80 and CD86 share receptors and can have comparable effects, several studies have demonstrated that these molecules can orchestrate distinctly different T cell functions [28], [29]. These non-redundant functions may reflect differences in binding affinities for their receptors and/or differences in pattern or distribution of ligand and receptor display at any given point during the T cell response [30]. Importantly, we did not see any impact of day 9p.i. CD86 blockade on the tempo of virus clearance as determined either by infectious virus titer or viral genome copy number (Figure 1C–D), consistent with an earlier report from our lab showing that CD86 blockade early during infection (i.e. day 5 p.i.) did not affect virus clearance [17]. This data suggested a unique role for CD86 during the recovery phase of infection following IAV clearance, which is distinct from its previously defined roles during the induction and effector phases of T cell responses.

### Innate pulmonary inflammation is increased after CD86 blockade

**Figure 2:**
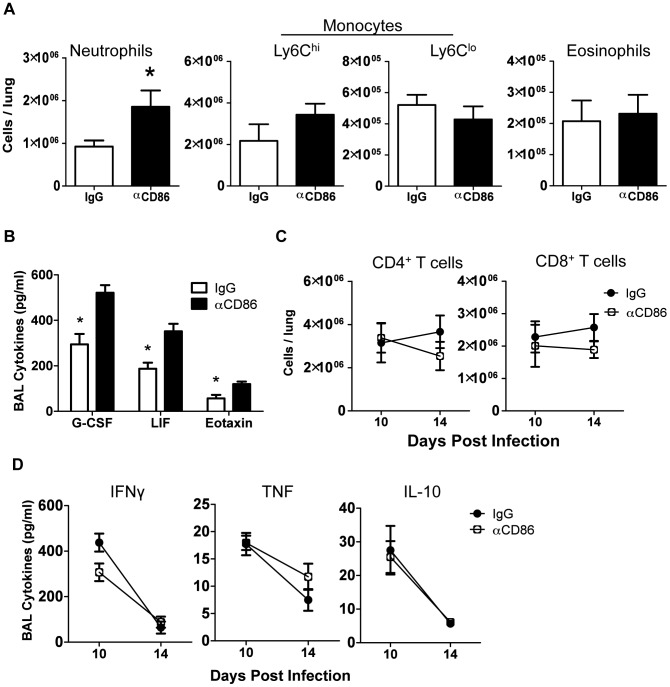
Innate pulmonary inflammation is increased after CD86 blockade. Balb/c mice were infected with 0.1 LD_50_ PR8 and treated with 200 µg αCD86 i.p. on day 9 p.i. (A) Innate immune populations were analyzed from lung cell suspensions using flow cytometry on day 14 p.i. Neutrophils were Ly6G^+^CD11b^+^, monocytes were Ly6G^−^CD11b^+^, and eosinophils were SiglecF^+^CD11b^+^CD11c^−^ (n = 7–10, combined from 3 independent experiments). (B) BAL from day 14 p.i. was analyzed by Luminex (n = 3–5, combined from 2 independent experiments). (C) Total CD4^+^ and CD8^+^ T cell responses were quantified from lung cell suspensions using flow cytometry (n = 7–10, combined from 3 independent experiments). (D) BAL from day 14 p.i. was analyzed for cytokines by Luminex (n = 3–5, combined from 2 independent experiments).

Since CD86 blockade led to increased morbidity without any change in viral clearance, we wanted to determine if CD86 blockade during the resolution phase of infection delayed recovery by affecting the extent or characteristics of pulmonary inflammation. In order to assess changes to immune responses after αCD86 treatment during the recovery phase (on day 9 p.i.), we sampled the bronchial alveolar lavage (BAL) fluid and immune cells within the respiratory tract at various times post-CD86 blockade. We detected an increase in the accumulation of neutrophils in the lung interstitium following CD86 blockade. However, the percentage and absolute numbers of other abundant lung infiltrating innate immune cells including Ly6C^hi^ monocytes, Ly6C^lo^ monocytes, and eosinophils were unchanged after αCD86 administration (Figure 2A). By Luminex 30-plex cytokine array analysis, we detected elevated levels of only three innate immune associated cytokines, G-CSF, LIF, and eotaxin in the BAL fluid following blockade (Figure 2B). All of these cytokines have chemotactic potential, and G-CSF, in particular, is associated with the capacity to support the generation and survival of neutrophils [31]. We did not detect any significant impact of CD86 blockade on total lung CD4^+^ or CD8^+^ T cell numbers within the IAV infected lungs, nor on BAL cytokines typical of effector T cell origin (e.g. IFNγ) (Figure 2C–D). If anything, there was a trend toward fewer CD4^+^ and CD8^+^ T cells, which is counterintuitive in mice with increased morbidity and inflammation. To further probe the potential impact of αCD86 treatment on antigen-specific T cells, we prepared lung cell suspensions from day 12 p.i. infected mice that had received αCD86 treatment on day 9 p.i., and we stimulated these cells ex vivo with PR8-infected BMDCs. Flow cytometry analysis of intracellular cytokine staining revealed that in vivo αCD86 blockade had no impact on the quantity of antigen specific T cells capable of producing IFNγ (Figure S1), suggesting that antigen-specific effector T cells were not overtly altered by αCD86 treatment during the recovery phase.

### Cellular display of CD86 and its receptors CTLA4 and CD28 in the lungs during resolution of IAV infection

**Figure 3:**
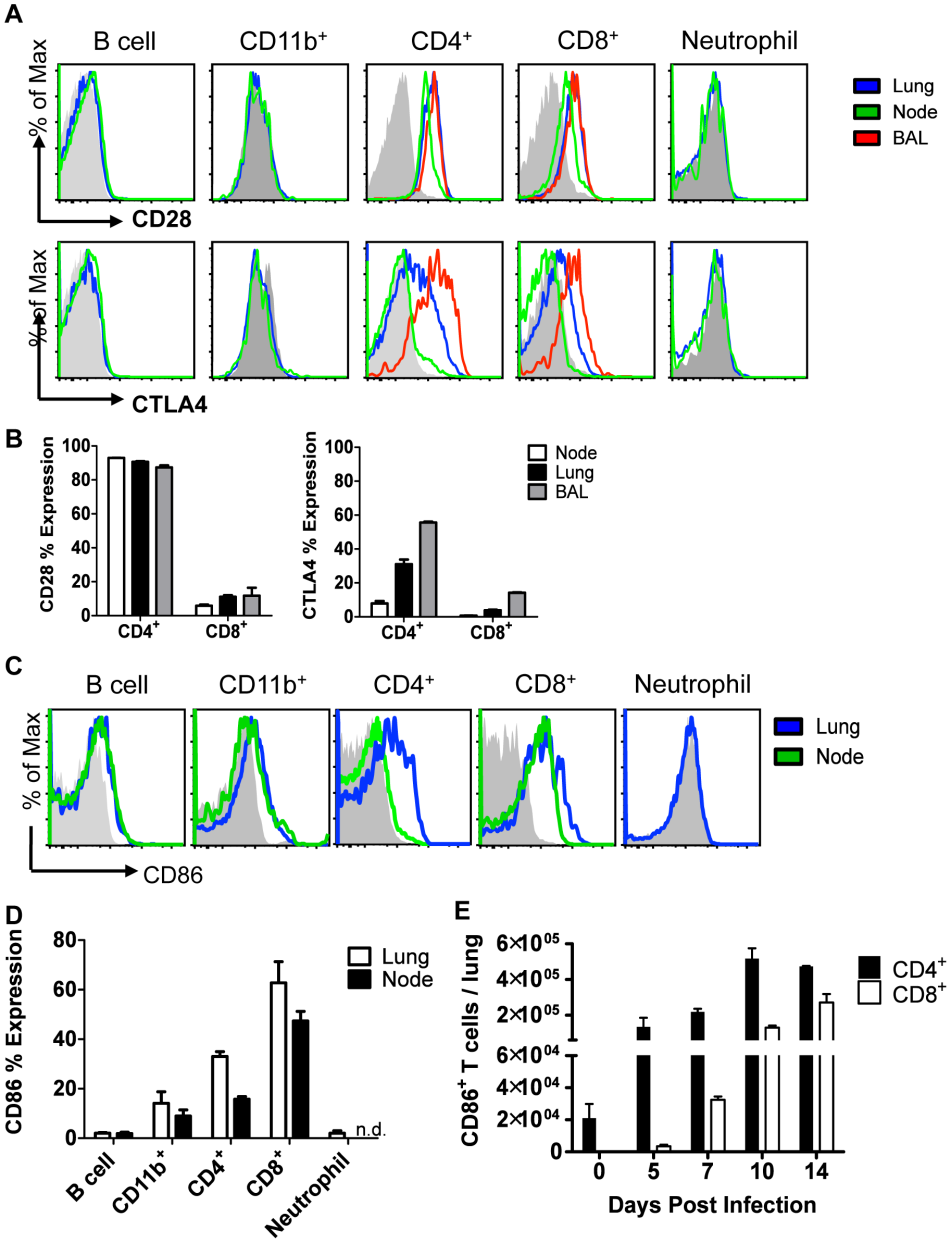
Cellular display of CD86 and its receptors CTLA4 and CD28 during resolution of IAV infection. Balb/c mice were infected with 0.1 LD_50_ PR8, and single cell suspensions were harvested from lung, draining lymph node, or BAL on day 10 p.i. (unless indicated otherwise) and analyzed by flow cytometry. (A–B) Cells were stained for surface CD28 or permeablized and stained for CTLA4. (C–D) Cells were stained for surface CD86 expression (n.d. = not done). (E) Lung cells were harvested at various days p.i., and surface CD86 expression was analyzed on CD4^+^ and CD8^+^ Thy1.2^+^ T cells.(A–E: n = 2, representative of 2 independent experiments).

To identify the potential cellular targets of CD86 blockade, we evaluated expression of CD86 and its receptors, CD28 and CTLA4, within the respiratory tract and the lung draining lymph nodes at day 10 p.i. We found that expression of CD28 and CTLA4, as expected, was restricted to T cells, with CD4^+^ T cells having the highest frequency of receptor-positive cells (Figure 3A–B). In contrast, CD86 had very promiscuous expression, and in addition to its display on the surface of traditional antigen presenting cells, including CD11b^+^ monocytes/macrophages and B220^+^ B cells, CD86 was also abundantly expressed on T cells in the recovering lungs (Figure 3C–D). The numbers of CD86^+^ CD4^+^ and CD8^+^ T cells increased during the course of infection, with maximal numbers around day 10 p.i. (Figure 3E). It is perhaps noteworthy that peak expression of CD86 includes the timeframe when in vivo blockade of this co-stimulatory ligand is most effective in promoting excess morbidity. Importantly, neutrophils expressed neither the CD86 ligand nor its receptors at this time point. This suggests that the increase in lung neutrophil accumulations observed after CD86 blockade is not due to a direct effect of blockade on the migration, accumulation or function of the neutrophils infiltrating the lungs. In view of the T cell-restricted expression of the CD86 receptors, CD28 and CTLA-4, it was likely that CD86 blockade was interfering with CTLA4 and/or CD28 signaling in T cells following virus clearance, ultimately leading to increased inflammation and increased neutrophil accumulation during the recovery phase of infection.

### CD86 blockade leads to selective Treg depletion

**Figure 4:**
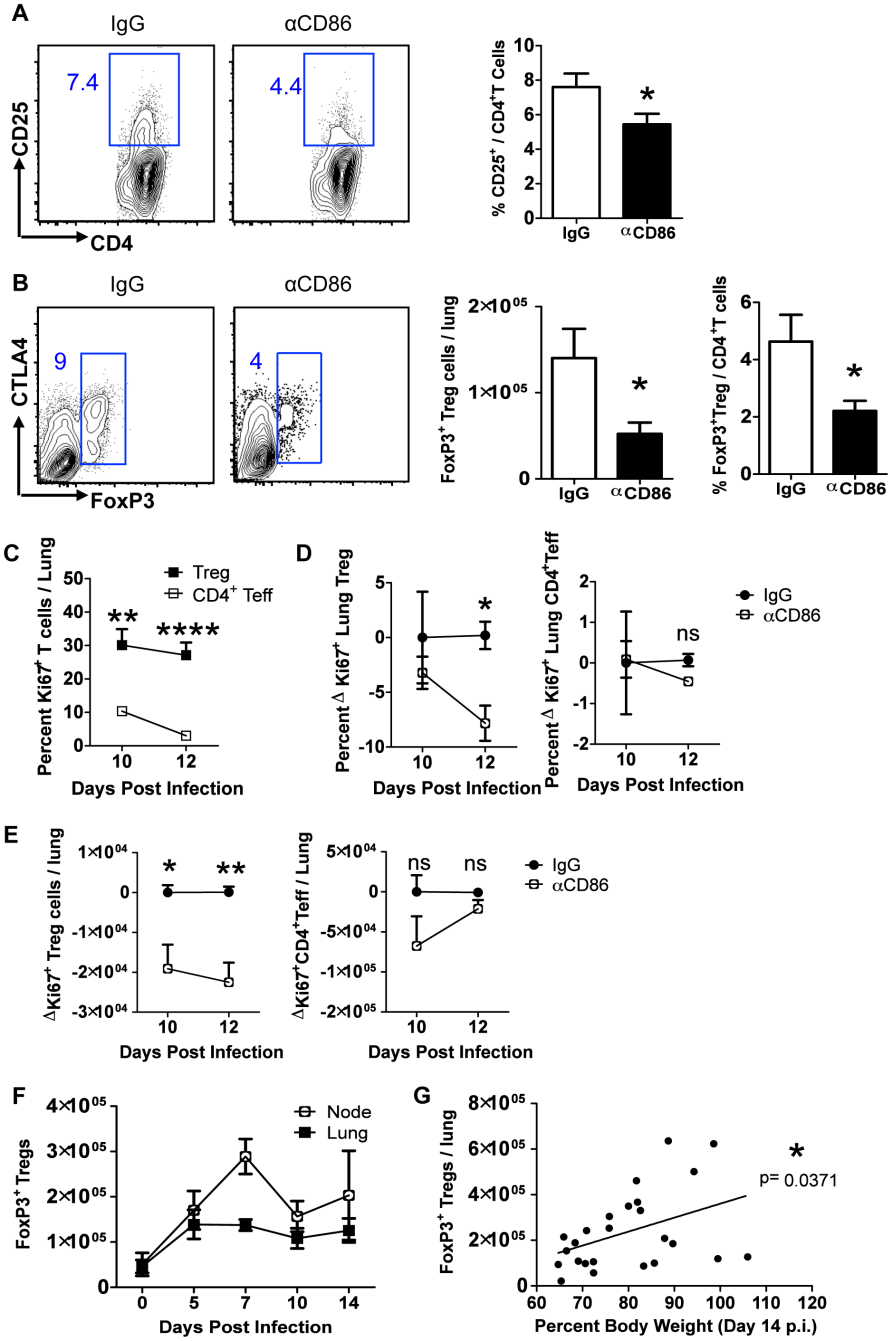
CD86 blockade leads to selective Treg depletion. Balb/c mice were infected with 0.1 LD_50_ PR8 and (A–B)treated with 200 µg αCD86 i.p. on day 9 p.i. Lung cell suspensions were harvested and analyzed by flow cytometry on days 10 or 14 p.i. (A) CD25 expression on CD4^+^ T cells harvested from lung on day 10 p.i. Representative flow plots and quantification are shown (n = 12, combined from 4 independent experiments). (B) Treg cells in lung cell suspensions harvested on day 14 p.i. Representative flow plots and quantification are shown (n = 5–8, combined from 3 independent experiments). (C) Ki67^+^ lung T cells on indicated day post infection. (D–E) Influenza-infected mice were treated with 200 µg isotype antibody (IgG) or αCD86 on day 9 p.i. (D) The percent and (E) absolute numbers of Ki-67^+^ cells were examined on indicated day post infection. The change in Ki67 expression (ΔKi67) was calculated with respect to the difference between the isotype control (considered 0) and the αCD86 treated animals. (C–E) Tregs were identified as Thy^+^CD4^+^FoxP3^+^, and Teff were identified as Thy^+^CD4^+^FoxP3^−^. Values considered statistically significant at *P<.05; **P<.01; ****P<.0001 (2 independent experiments; 2–3 mice/experiment). (F) Lung and draining lymph node cell suspensions were analyzed for Thy1.2^+^ CD4^+^ FoxP3^+^Tregs by flow cytometry after influenza infection (n = 4–9, combined from 3 independent experiments). (G) IgG treated Balb/c mice or saline treated DEREG mice were infected with PR8 and harvested on day 14 p.i. FoxP3^+^Tregs from lung were analyzed by flow cytometry (combined from 10 independent experiments).

Although we observed no change in overall T cell numbers in the respiratory tract following CD86 blockade in vivo (Figure 2C), we did observe that there was a transient but significant decrease in CD25 expression on lung CD4^+^ T cells one day following CD86 blockade, that is on day 10 p.i. following CD86 blockade on day 9 p.i. (Figure 4A). Since CD25 serves as a marker for regulatory T cells (as well as activated effector T cells), we evaluated the impact of in vivo CD86 blockade on Treg cells in the lungs by flow cytometry (using FoxP3^+^ expression as a marker for the Treg cell compartment). We found a significant reduction in both the frequency and absolute number of CD4^+^Treg cells in the lungs on day 14 p.i. after CD86 blockade administered on day 9 p.i. (Figure 4B). This could explain the slight trend toward lower CD4^+^ T cells seen in Figure 2 since FoxP3^+^Treg cells are embedded in that data. Interestingly, although we saw a decrease in CD25 expression on total CD4^+^ T cells at day 10 (one day after CD86 blockade), we did not see a reduction in CD25 expression on the remaining Tregs cells on day 14 p.i., suggesting that although Tregs were diminished in numbers, their per cell activation state was not altered by αCD86 blockade (Figure S2A). To further evaluate Treg function after CD86 blockade, we harvested lung cell suspensions from day 12 p.i. mice (that had received αCD86 on day 9), used PR8 infected BMDCs to re-stimulate lung T cells, and then measured IL-10 production in FoxP3^+^Tregs by intracellular cytokine staining. We found that although the total percentage of Tregs was beginning to drop within the total CD4^+^ population (Figure S2B), the frequency of IL-10^+^ Treg cells was not reduced within the FoxP3^+^ population (Figure S2C), suggesting that Treg function in remaining cells was not altered by αCD86. Importantly, using the same day 12 p.i. lung cell suspensions, we included αCD86 in vitro in separate BMDC co-cultures to evaluate if the presence of the blocking antibody in the cultures could reduce Treg IL-10 production. We saw no impact of αCD86 on the frequency of IL-10^+^Tregs under these culture conditions, further supporting the idea that αCD86 did not directly alter Treg function on a per cell basis (Figure S2D).

We next wanted to determine if the Treg cells in the infected respiratory tract expressed CD86 and/or its receptors CD28 and CTLA4. We found that Tregs did express high levels of both CD28 and CTLA4, as well as lower levels of CD86 over the course of infection (Figure S3 A–C). These data suggest that CD86 blockade could prevent signaling on Treg cells through CD28 and/or CTLA4, which are required for Treg homeostatic maintenance and proliferative expansion [13]. It is unlikely CD86 blockade affected Tregs intrinsically since the majority of Treg cells in the respiratory tract were CD86 negative, and there is no known signaling role for CD86 on T cells. Because CD28 and CTLA4 can promote Treg proliferation, we wanted to determine whether CD86 treatment caused a defect in Treg proliferation, which could plausibly explain the loss of Treg numbers in the respiratory tract. We examined Ki67 expression in both conventional CD4^+^FoxP3^−^ T effector cells and FoxP3^+^Treg cells in the lung after CD86 blockade (administered at day 9 p.i.). We found that a sizable percentage of Tregs in the recovering lung were Ki67^+^, and this high percentage was largely maintained from day 10 to day 12 p.i. In contrast, the CD4^+^ effector T cells had a much lower frequency of proliferating cells that continued to drop to near baseline from day 10 to day 12 p.i. (Figure 4C). Importantly, when we compared the frequency and numbers of proliferating Tregs and CD4^+^ effector T cells in the lung after αCD86 treatment, we saw a significant decrease in the quantity of Ki67^+^Tregs, suggesting that CD86 co-stimulation promotes Treg proliferation after IAV infection (Figure 4D–E). There was a trend toward reduced Ki67^+^ effector CD4^+^ T cell proliferation after αCD86 treatment, but this did not reach statistical significance, likely because only a small fraction of these cells were proliferating at this late time after infection. This observation is consistent with the previous findings from our lab that the effector T response rapidly wanes after day 7 p.i. in the absence of influenza antigen [17], and it provides a plausible explanation as to why the late CD86 blockade selectively impacts Treg cells, not T effector cells.

Since Treg cells play an anti-inflammatory role in many disease models, a loss of Treg cells following in vivo CD86 blockade could, in part at least, explain the observed increase in lung inflammation. Treg cells have been reported to suppress CD8^+^ T cell responses as well as innate immune responses at the induction of IAV-specific immune responses or during IAV clearance [23], [25], [32]; however, to our knowledge, no role for Treg cells has been established during the recovery phase of IAV infection, that is after infectious virus clearance.

To explore the potential role of Treg cells in the resolution of inflammation during recovery from influenza infection, we first determined the kinetics of Treg cell accumulation in the respiratory tract during IAV infection. To this end, we infected wild type mice with IAV and determined the number of Tregs in the lungs and draining lymph nodes at various times p.i. Consistent with earlier reports [25], [33], we found that Treg cells are recruited to and accumulate in the lungs following infection, and, of note, Treg cell numbers also persist well into the recovery phase (Figure 4F). Consequently, these cells have the potential to participate in limiting excess residual inflammation even after infectious virus has been cleared and virus-infected cells largely eliminated. Furthermore, we found that even in wild type infected mice not undergoing CD86 blockade the number of Treg cells detected in the lung after virus clearance positively correlated with recovery of body weight (Figure 4G). This difference in Treg numbers among infected untreated wild type animals could, in part, contribute to the variability in tempo of weight gain that is typically observed during the recovery/resolution phase of IAV infection.

### Treg cells promote recovery after IAV infection by limiting innate inflammation

**Figure 5:**
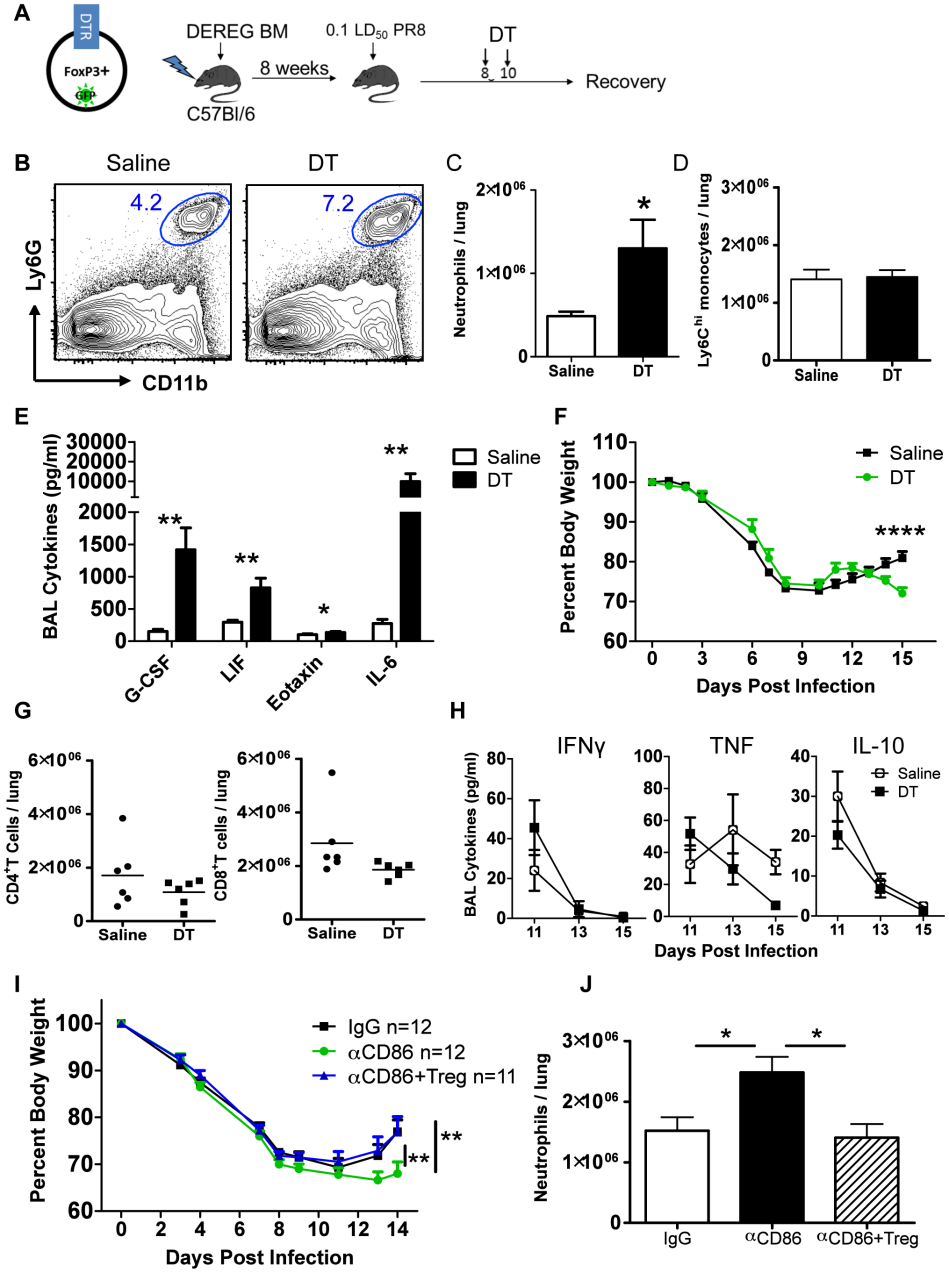
Treg cells promote recovery after IAV infection by limiting innate inflammation. (A) Schematic for generation and infection of DEREG bone marrow chimeras. DEREG bone marrow chimeras were infected with 0.1 LD_50_ PR8 and 40 µg/kg diphtheria toxin (DT) was administered i.p. at days 8 and 10 p.i. (B) Representative flow cytometry plots of lung cell suspensions harvested on day 15 p.i. (C–D) Quantification of total numbers of lung Ly6G^+^ neutrophils and CD11b^+^Ly6G^−^Ly6C^hi^ monocytes harvested day 15 p.i. (n = 6). (E) Day 15 p.i. BAL was analyzed for cytokines by Luminex (n = 6). (F) Weight loss in DEREG BM chimeras (n = 6). (G) Flow cytometry of lung cell suspensions harvested on day 15 p.i. (H) BAL cytokines after Treg depletion were measured by Luminex (n = 4–6). All data in B–H are combined from 3 independent experiments. (I–J) Balb/c mice were infected with 0.1 LD_50_ PR8, treated with 200 µg αCD86 on day 9 p.i. then received i.v. transfer of 2×10^6^Tregs on day 11 p.i. (combined from 4 independent experiments) (J) Lung cell suspensions were harvested on day 14 p.i. and analyzed by flow cytometry (n = 6–7, combined from 2 independent experiments).

The above findings suggested that one effect of in vivo CD86 blockade was to limit the accumulation and/or persistence of Treg cells necessary to limit inflammation following IAV clearance from the lung. We therefore wanted to independently evaluate the role of Tregs during recovery and in particular whether depletion of Treg cells in vivo at this late time point following infection (i.e. day 8–10 p.i.) would mimic the effect of CD86 blockade. To explore this possibility, we employed the DEREG (Depletion of REGulatory T cells) mouse model to deplete Tregs after virus clearance but before the onset of recovery. DEREG mice express the diphtheria toxin (DT) receptor (and GFP) driven off of the FoxP3 promoter [34]. Because of reports indicating that FoxP3 may be expressed on cell types other than Tregs, in particular epithelial cells from multiple organs, including the lung [35] and to ensure that DT-mediated depletion only affected immune cells, for this analysis we employed bone marrow chimeric mice in which DEREG bone marrow was used to reconstitute irradiated wild type animals (Figure 5A). Following irradiation and reconstitution, we infected these mice and administered DT at days 8 and 10 p.i. We found that DT administration lead to a rapid decrease in total Treg cells in the lungs (Figure S4A). While loss of GFP^+^ Treg cells was extensive and prolonged, the small number of GFP^−^Tregs did preferentially expand following DT treatment ultimately resulting in the restoration of total Treg numbers within one week (Figure S4A). We evaluated innate immune responses in the respiratory tract after Treg depletion and found that Treg depletion in DEREG mice mimicked the inflammatory signature of CD86 blockade at days 8–10 p.i. There was a significant increase in neutrophil numbers (Figure 5B–C), but not Ly6C^hi^ monocytes (Figure 5D), and the innate cytokines G-CSF, eotaxin, and LIF were also increased in the airways (BAL fluid) (Figure 5E). Also similar to the CD86 blockade model, Treg cell depletion delayed regain of weight in mice (Figure 5F), without changing the kinetics of virus clearance (Figure S4B). Furthermore, although effector T cells have been implicated as targets of Treg suppression early during infection [23], late Treg cell depletion had no impact on the total numbers of conventional CD4^+^ and CD8^+^ T cells in the lungs (Figure 5G), or the levels of prototypical effector T cell cytokines (e.g. IL-10, IFN-γ, and TNF) in the BAL fluid (Figure 5H).

Treg depletion did not completely mimic the effect of CD86 blockade. For example, we noted a large increase in the innate cytokine IL-6 following acute Treg cell depletion (Figure 5E) which was not detected following CD86 blockade. Also, the defect in weight gain was delayed in the DEREG model compared to the αCD86 model, but this defect coincided with the time post-CD86 blockade when Treg numbers are detectably reduced. We did not, however, carry out an extended time course in these mice to determine when or if Treg depleted mice eventually recover full body weight. Thus the αCD86 blockade and Treg depletion models showed significant overlap, but unlike αCD86 treatment which produced a gradual decrease in lung Treg cell numbers over several days, acute Treg depletion produced by DT administration likely had additional consequences (e.g. elevated IL-6 production).

To determine if the delay in recovery and excess inflammation triggered by CD86 blockade was at least in part attributable to the loss of Treg cells in the lungs following CD86 blockade, we administered αCD86 to infected mice on day 9 p.i., and then adoptively transferred 2×10^6^Tregs by the i.v. route on day 11 p.i. We found that the Treg transfer following CD86 blockade lead to accelerated weight gain (Figure 5I). Importantly, we observed a decrease in total neutrophils in the respiratory tract (Figure 5J) following Treg cell transfer. These findings suggest that loss of Tregs resulting from CD86 blockade contributed to delay in recovery from IAV infection, and that following infectious virus clearance, Treg cells may play a critical role in controlling residual inflammation and facilitating recovery from infection.

We chose this CD86 blockade and transfer schedule because the αCD86 antibody potently masks the CD86 epitope for only approximately 48 hours post injection in the respiratory tract (data not shown). Therefore, transferring cells two days post αCD86 treatment minimizes effects of residual antibody on the transferred Treg cells, although CD86 blockade did not suppress Treg function as measured by CD25 expression and IL-10 production (Figure S2) and the majority of isolated Treg cells did not express CD86 (Figure S5). However, since some transferred Tregs cells, isolated from naïve spleen, did express CD86, we cannot completely rule out a possible functional contribution of CD86 expressed on Tregs in facilitating recovery from influenza virus infection.

### Treg cells suppress neutrophil-dependent cytokines

**Figure 6:**
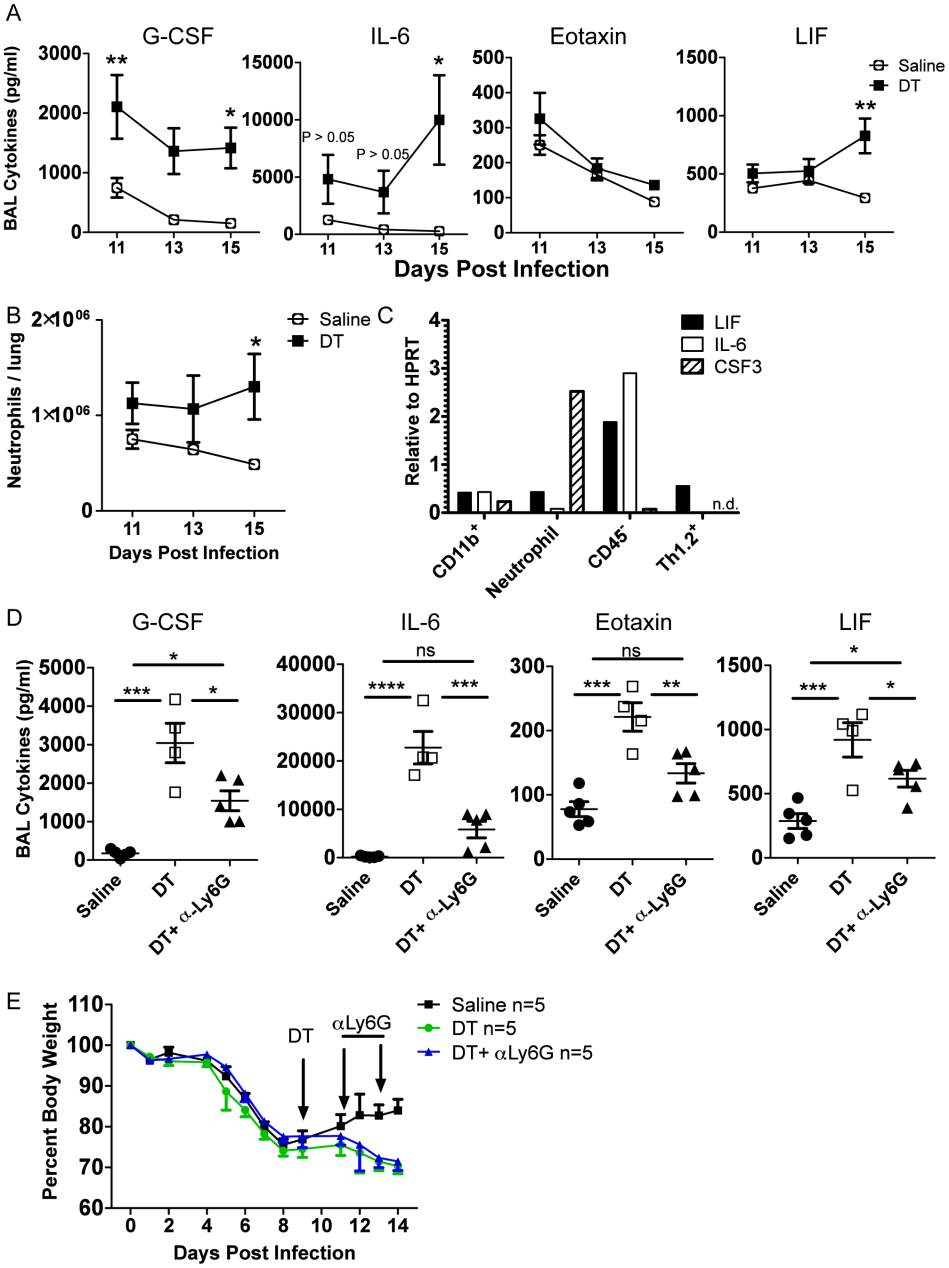
Tregs suppress neutrophil-dependent cytokines. DEREG bone marrow chimeras were infected with 0.05 LD_50_ PR8. (A) BAL cytokines were analyzed by Luminex and (B) lung neutrophils were analyzed by flow cytometry at various days post depletion (A–B: n = 4–5, combined from 2 independent experiments). (C) WT C57BL/6 mice were infected with PR8 and qRT-PCR was done on RNA from flow cytometry-sorted lung cell populations harvested on day 15 p.i. “CD11b^+^” cells were CD11b^+^Ly6G^−^, and neutrophils were CD11b^+^Ly6G^+^ (n.d. = not detected). (D–E) DEREG mice were infected with 0.05 LD_50_ PR8, 40 ug/kg DT was injected on day 9 p.i., and 50 ug αLy6G neutrophil depleting antibody (1A8) was administered i.p. on days 11 and 13 p.i. (D) Day 14 BAL cytokines were analyzed by Luminex (n = 4–5). (E) Weight loss (n = 5). Data in D–E are combined from 2 independent experiments.

To determine the possible direct targets of Treg suppression during recovery, we took a closer look at the kinetics of inflammatory cells and mediators after Treg depletion in DEREG mice, this time using a single dose of DT at day 9 p.i. We found that IL-6, LIF, and neutrophil numbers were not statistically significantly impacted until several days after Treg depletion, suggesting that they were not directly suppressed by Tregs (Figure 6A–B), although there was a trend that IL-6 appeared to be elevated shortly after Treg depletion. Importantly, G-CSF was immediately and dramatically increased, suggesting that the cellular source(s) of G-CSF was under direct Treg control (Figure 6A). We analyzed the mRNA content of various cell populations from the lung on day 15 p.i. and found that the majority of *CSF3* mRNA was found within the neutrophil compartment, with lower but still detectable levels in the CD45^−^ and macrophage/monocyte compartments (Figure 6C). This suggests that Tregs may directly suppress neutrophil-derived G-CSF, which could contribute to pulmonary inflammation through a feed-forward circuit of neutrophil recruitment and/or persistence in the respiratory tract.

Since neutrophils were identified as a potential source of G-CSF and target of Treg suppression, we wanted to determine if neutrophil depletion would rescue the effects of Treg depletion. We used the neutrophil-depleting αLy6G (1A8) antibody to reduce the number of neutrophils after Treg depletion. Using the DEREG bone marrow chimeras, we infected mice with a sublethal dose of PR8, injected DT at day 9 p.i., and administered 50 µg of αLy6G mAb on days 11 and 13 p.i. We then measured parameters of lung inflammation on day 14. We found that neutrophil depletion significantly reduced excess cytokines in the BAL after Treg depletion, suggesting that neutrophils are a major source or stimulator of inflammatory cytokines after Treg depletion (Figure 6D). Neutrophil depletion failed, however, to rescue weight loss after Treg depletion (Figure 6E), suggesting that Treg cells may regulate other features or aspects of the recovery process following IAV clearance. Alternatively, subsets of neutrophils exist that can possess anti-inflammatory functions; therefore, total neutrophil depletion may lead to loss of both pro-inflammatory neutrophils and putative beneficial neutrophils.

## Discussion

In this report, we described a previously unappreciated positive role for the co-stimulatory ligand CD86 in the resolution of inflammation following virus clearance during recovery from IAV infection. We observed that blocking CD86 (but not CD80) engagement in vivo in a narrow time window (i.e. between days 8 and 12 p.i.) resulted in prolonged morbidity (i.e. delayed regain of weight). This delay in recovery was associated with a sustained innate inflammatory response reflected in elevated levels of several cytokines/chemokines in the BAL fluid and excess accumulation of neutrophils in the lung tissue compared to the experimental controls. This “late” blockade of CD86 in vivo did not alter the frequency or activity of activated CD8^+^ and CD4^+^ T effector cells present in the recovering lungs; however; CD86 blockade did result in a significant decrease in the FoxP3^+^ Treg cell population present in the respiratory tract, due at least in part to a reduction in the proliferation of Treg cells. Targeted Treg cell depletion during the aforementioned time late in infection in the DEREG mouse model mimicked the inflammatory signature of the CD86 blockade phenotype, and adoptive transfer of Treg cells, in part, rescued the effects of the CD86 blockade. Finally, we identified neutrophils as a target for regulation/suppression by Treg cells since neutrophil depletion reversed the effect of Treg cell depletion on inflammatory cytokines in the BAL.

In the present study, we demonstrated a novel role for the co-stimulatory B7 family member CD86 to resolve ongoing inflammation following IAV clearance through maintenance of the Treg cell response. Resolution is defined as a return to homeostasis, and this process encompasses both the dampening of the immune system and initiation and completion of repair processes, ultimately leading to the regain of normal physiological function [7], [10]. Previous studies investigating the co-stimulatory B7 family molecules during influenza infection have demonstrated their critical role in activating naïve T cells in the draining lymph nodes and triggering IAV-specific T cell effector activity within the respiratory tract [15], [16], [36]. In the context of both induction and the expression of effector T cell responses to influenza infection, CD80 and CD86 appeared to have overlapping, essentially equivalent roles acting in concert with viral antigen presented on the surface of an APC to trigger naïve T cell activation and/or expression of T cell effector activity [17]. However, our report indicated that during the recovery/resolution phase of IAV infection, CD86 displayed a unique activity (i.e. not displayed by CD80) to control excess inflammation in the lungs following virus clearance without any apparent impact of this co-stimulatory ligand on residual lung effector T cells.

Non-redundant roles for CD80 and CD86 have been reported in other disease models where these two co-stimulatory molecules have been demonstrated to display different roles in the promotion of responses by either Treg cells or T effector cells. This phenomenon has been most well-studied in the NOD mouse model of type I diabetes. Counter to what we observed in the IAV infection model, in NOD mice CD80 is critically important for Treg maintenance, whereas CD86 promotes the autoreactive effector T cell response [19], [37]. However in other models, CD86, not CD80, has been demonstrated to preferentially promote Treg responses [29], [38]. In a comprehensive review of the regulation of Treg cells by CD80/86 co-stimulation, Bour-Jordan et al. argues that these seemingly contradictory findings were likely due to differences in expression levels of CD80 and CD86 in both time and space across different model systems [13]. As we demonstrate in this report, in the IAV infection model, CD86 plays a unique role in controlling excess inflammation following virus clearance through its ability to sustain Treg cell numbers and/or function.

To our knowledge, this study is the first to evaluate the role of Tregs during resolution of influenza-induced disease, although Treg cells have been implicated in inflammation/injury resolution in models of acute lung injury (ALI) [26], [27]. Models of ALI share certain characteristics with influenza-induced lung injury, including infiltration of neutrophils and other leukocytes into the respiratory tract, epithelial damage, increased vascular permeability, and hypoxemia. In ALI, Treg cells become activated by either pattern recognition receptors (e.g. TLR4 for LPS-induced ALI) or injury-related self-antigens to promote neutrophil apoptosis and reduce cytokine production by phagocytic macrophages [11], [39]. A similar environment potentially exists during the recovery phase after IAV clearance: viral antigen is low, injury-related self-antigens are abundant, and Treg cells are required to limit neutrophil accumulation and innate cytokine production in the respiratory tract. It remains to be determined how Treg cells function to limit neutrophil numbers and promote weight gain after influenza infection, but it may be possible to draw parallels with the ALI model, where Treg-derived TGF beta and adenosine (through the ecto-enzyme CD73) have been shown to diminish inflammation and promote resolution of injury [11], [27].

An increasing body of evidence and emerging consensus suggests that IL-10 is not a major mechanism by which Tregs promote resolution of lung injury [39], although Tregs are capable of producing IL-10 during the resolution phase. This view is consistent with our findings on the level of IL-10 expression during the recovery/resolution phase of influenza infection. IL-10 is an important anti-inflammatory molecule during virus clearance by effector T cells and is primarily produced by effector T cells themselves [40], but as we showed in Figure 2, the level of IL-10 in the BAL during the recovery phase is very low and does not depend on Tregs.

It is also notable that effector T cells do not appear to be targets of Treg suppression during the recovery phase after IAV infection, in contrast to a previously described role for Tregs to suppress the CD8^+^ T cell response during the induction phase of IAV infection. It is likely that at this late stage after infection, IAV antigen availability limits the T cell responses without the need for Treg suppression. This concept is supported by previous work from our lab: Hufford et al. showed that the in vivo CD8^+^ T cell IFNγ response, which is dependent on antigen presentation, decreased dramatically from day 7 to day 8 p.i., correlating with clearance of infectious virus from the BAL fluid [17]. Therefore, it appears that by day 8 or 9 post infection, at the time of Treg depletion, effector T cell responses are already past their peak and are likely regulated by antigen availability, with no requirement for control by Tregs.

The mechanism(s) by which CD86 sustains Treg proliferation and total Treg numbers during recovery from IAV infection still needs to be more fully defined, but several possibilities can be considered. CD28 and CTLA4 are expressed on Treg cells, and these receptors have been shown to be critical for Treg development, homeostasis, proliferation, and function [13], [41]. Consequently, disruption of CD86 ligand engagement for one or both of these receptors directly on Treg cells could result in reduced proliferation, adversely affecting the numbers of Treg cells as suggested by our findings. However, additional studies would be required to determine if CD86 blockade could be impacting Treg survival and/or function in addition to proliferation. Alternatively, although we did not detect any changes in prototypical IAV-specific T cell IFNγ production, it is possible that αCD86 treatment could impact the Treg response indirectly through other less well studied effector T cell derived functions. For example IL-2,a critical regulator of Treg viability and maintenance, could potentially help sustain Treg responses in the late phase of influenza infection, with CD4^+^ T effector cells serving as a source of IL-2 through a CD86 engagement dependent mechanism [42], [43]. It is important to note that although there is some evidence that CD86 itself can signal upon ligation with its receptors in certain contexts on dendritic cells and B cells [30], [44], CD86 has not been described to signal in T cells and has no known signaling domain, suggesting that αCD86 likely does not impact T cells through blockade of an intrinsic CD86-derived signal.

It is important to acknowledge that although we see dramatic effects of αCD86 blockade in the lung, we cannot rule out that the antibody may be acting in another location (e.g. draining lymph node) to reduce Treg cell numbers, ultimately resulting in loss of Treg cells in the lung and enhanced inflammation in the respiratory tract. However, although the blockade is not restricted to the lung, the blocking antibody does at least block CD86 in the respiratory tract, since labeled αCD86 does not bind to lung cells harvested from mice within 48 post in-vivo blockade, indicating that CD86 epitope is masked. Due to high levels of capillary leak and alveolar injury at the time of administration (day 10 post infection), lung specific blockade of CD86 would not be practicable since intranasal administration of αCD86 would readily enter the systemic circulation.

One interesting observation from this report is the expression of CD86 on both Teff (Figure 3C–E) and Treg cells (Figure S3) during the recovery phase. Although it is unclear if CD86 on T cells contributes to Treg maintenance in our system, Taylor et al reported that T cell-T cell interactions (i.e. CD86 on T cells triggering receptors on other T cells) may preferentially occur through CD86, and these interactions may be important to ameliorate disease in a model of graft-versus-host disease [45]. Alternatively, Tregs may require CD86 in the context of a classic APC, and we have observed that MHC II^+^ mononuclear cells in the respiratory tract late after infection may express either CD80 or CD86 or both (data not shown). Furthermore, differences in distribution of CD80 and CD86 expression on various APCs, including B cells, monocytes/macrophages, and dendritic cells, may explain why CD86 alone promotes resolution of disease in this model. Finally, if Treg cells are indeed responding to injury derived self-antigens and/or other damage associated signals to promote resolution of inflammation after IAV clearance, then CD86 may be critical to provide co-stimulation for these self-specific responses. Yadav et al demonstrated that CD86 preferentially primed self-antigen specific Teff and Treg cells in the NOD model, and CD80 was unable to compensate for this function in CD86 KO mice [37]. Unfortunately, dissecting the critical cellular source of CD86 during recovery is difficult because we would need to target CD86 disruption in both time (recovery period) and space (specific cell populations), but these questions could possibly be answered with the development of a cell-specific inducible CD86 knock out mouse. Understanding the cellular interactions required for CD86-mediated recovery and Treg cell maintenance would provide more targets to manipulate this novel pro-resolution pathway.

The contribution of neutrophils to inflammation and disease during influenza infection is complex and controversial [46]–[48]. Our data suggests that in the absence of Treg cells, lung infiltrating neutrophils may be detrimental during the recovery phase after influenza infection possibly by producing and/or by promoting release of pro-inflammatory cytokines by other cell types (e.g. respiratory epithelial cells) into the recovering lungs. This possibility is supported by evidence that neutrophils express a detrimental inflammatory signature during highly pathogenic influenza infections [49] and that Treg cells suppress innate cell dependent inflammation in influenza infected Rag^−/−^ mice [32].

The mechanism by which neutrophils promote cytokine production and how this impacts overall recovery after virus infection remains to be defined. Because we found that neutrophils expressed high mRNA levels of the pro-survival cytokine G-CSF, one hypothesis is that neutrophil accumulation fuels a feed-forward inflammatory circuit, and continued inflammatory functions of neutrophils drive cytokine production by nearby immune and epithelial cells. However, neutrophils are capable of pro-resolution functions in addition to their more well-characterized pro-inflammatory roles [50], [51], so it is possible that Tregs may promote a pro-resolution function in neutrophils, in addition to preventing accumulation of inflammatory neutrophils. This could possibly explain why neutrophil depletion did not rescue all aspects of recovery since neutrophil depletion may eliminate pro-resolution factors in addition to detrimental pro-inflammatory factors. Finally, even though other cell numbers in the respiratory tract were not increased, Tregs may target and functionally alter other cells during recovery, including dendritic cells, monocytes/macrophages, and CD45^−^ respiratory epithelial cells.

A potential alternative Treg target is the cellular source of IL-6. We observed an apparent increase in IL-6 immediately following Treg depletion although this trend did not reach statistical significance until 6 days after Treg depletion. Based on our gene expression analysis from sorted lung cells late after infection, BAL IL-6 could be derived from either CD11b^+^ monocytes/macrophages or CD45^−^ cells, which include lung epithelial cells, endothelial cells, and other stromal cells such as fibroblasts, and these cells could be novel targets of Treg suppression during the recovery phase. Furthermore, IL-6 is a potent pro-inflammatory cytokine which could have local effects to promote neutrophil survival in the lung [52], as well as systemic effects on eating/weight gain as it enters the circulation from the damaged respiratory tract [53], which could contribute to continued weight loss.

Furthermore, our studies of the resolution phase after IAV infection has been limited to analyses of cytokine mediators and inflammatory cells, but recent studies have highlighted the importance of lipid mediators (e.g. resolvins and lipoxins) [10] and growth factors (e.g. amphireggulin) [8] in the resolution of lung inflammation and injury. Future studies of Tregs in the control of resolution after respiratory infection should include analyses of other classes of soluble mediators, which would perhaps help identify alternative Treg targets. Interestingly, both growth factors and lipid mediators are largely derived from epithelial cells, which could represent a novel Treg target in resolution.

Taken together, this report describes a novel pro-resolution role for CD86 co-stimulation late after IAV infection. CD86 is required for a robust Treg response during the recovery phase after IAV clearance. Treg cells control the extent of pulmonary neutrophilia during the resolution phase of infection and regulate the expression of several cytokines of innate immune origin released into the recovering lungs. Treg cells promote resolution of inflammation, at least in part, by suppressing neutrophil-dependent cytokine production in the respiratory tract after virus clearance. Finally, since this late onset Treg cell response does not impact IAV clearance, the CD86-dependent Treg response could be a viable therapeutic target to suppress excess inflammation/injury without interfering with virus clearance.

## Materials and Methods

### Mice and infections

BALB/c, C57BL/6, and DEREG mice were purchased from the National Cancer Institute (NCI). All mice were housed at the University of Virginia in a pathogen-free environment. Mice used in experiments were between 8–12 weeks old and matched for age and sex. Type A influenza virus A/PR/8/34 (H1N1) was grown in day 10 chicken embryo allantoic cavities as described previously [54]. Mice were infected with 300 egg infectious doses (EID_50_) of A/PR/8/34 i.n. (corresponding to a 0.1 LD_50_ dose) unless otherwise noted. Treg cells were depleted from DEREG mice by administration of 40 ug/kg diphtheria toxin i.p. at the indicated day post influenza infection.

### Preparation of tissue and single-cell suspensions

Mice were sacrificed by cervical dislocation. Lungs were perfused through the right heart with 10 mL PBS to remove cells from the vasculature. To prepare single cell suspension, lungs were minced and digested in media containing 183 U/mL collagenase D (Worthington) for 45 minutes at 37°C. Lung tissue was then disrupted through a steel screen, and red blood cells were lysed with ACK lysis buffer. Live cells were determined by trypan blue exclusion and counted with a hemocytometer. To prepare total lung RNA, lungs were processed with an electric homogenizer in 1 mL TRIzol (Invitrogen) and stored at −80°C.

### qRT-PCR

RNA was extracted from TRIzol (Invitrogen) homogenized samples, and cDNA was generated using Superscript III (Life Technologies) according to the manufacturers' protocols. qPCR was performed on a Life Technologies StepOne instrument using SYBR Green (Life Technologies) according to manufacturer's instructions. Relative gene expression is calculated by the following formula: 2∧(ΔCt), where ΔCt = Ct(HPRT) - Ct(gene of interest). PCR primer sequences are as follows: Flu *PA* (Fw 5′-CGG TCC AAA TTC CTG CTGCTG A-3′ and Rev 5′-CAT TGG GTT CCT TCC ATC CA-3′), Flu *NP* (Fw 5′-AGG GTC GGT TGC TCA CAA GT-3′ and Rev 5′-TGC TGC CAT AAC GGT TGT TC-3′), *HPRT* (Fw 5′-CTC CGC CGG CTT CCT CCT CA-3′ and Rev 5′-ACC TGG TTC ATC ATC GCT AAT C-3′), *LIF* (Fw 5′-ATG TGC GCC TAA CAT GAC AG-3′ and Rev 5′-TAT GCG ACC ATC CGA TAC AG-3′), *CSF3* (Fw 5′-ATG GCT CAA CTT TCT GCC CAG-3′ and Rev 5′-CTG ACA GTG ACC AGG GGA AC-3′), *IL6* (Fw 5′-ACG GCC TTC CCT ACT TCA CA and Rev 5′-TCC AGA AGA CCA GAG GAA ATT TT-3′), and *Eotaxin* (Fw 5′-CAG ATG CAC CCT GAA AGC CAT A-3′ and Rev 5′-TGC TTT GTG GCA TCC TGG AC-3′).

### Antibodies for flow cytometry

The following mAbs were purchased from BD or eBioscience (unless otherwise stated), as conjugated to FITC, Alexa-488, PE, PE-Cy7, PerCP-Cy5.5, APC, Alexa Fluor 647, APC–Alexa Fluor 780, or biotin: CD4 (GK1.5), CD4 (L3T4), CD8α (53–6.7), CD11b (M1/70), CD11c (HL3), CD19 (1D3), CD25 (PC61), CD25 (7D4), CD45 (30-F11), CD80 (16-10A1), CD86 (GL-1), CD90.2 (53–2.1), Gr-1 (RB6-8C5), SigLecF (E50-2440), Ly6G (1A8), Ly6C (AL-21), I-A^d^ (AMS-32-1), CTLA-4 (UC10-4F10-11), FoxP3 (FJK-16s), and CD28 (37.51). Anti–mouse CD16/32 used for F_c_ receptor blocking was isolated and purified in the University of Virginia Hybridoma Core Facility.

### Flow cytometry analysis

Cells were suspended in buffer containing PBS, 2% FBS, 10 mM EDTA, and 0.01% NaN_3_. Fc receptors were blocked with anti-mouse CD16/32, and then cells were incubated with specific monoclonal antibodies or fluorescence minus one controls for 30 minutes at 4°C. Where indicated, after surface staining, intracellular FoxP3 staining was performed using the FoxP3 fixation/permeabilization kit (eBiosciences). Flow cytometry was performed on FACS Canto flow cytometers (BD), and data were analyzed using FlowJo (Tree Star, Inc.).

### BAL fluid and cytokine determination

Bronchial alveolar lavage (BAL) fluids were harvested by cannulating the trachea and injecting, then withdrawing 0.5 mL PBS into the airways three times. Cells were removed by centrifugation and supernatants were stored at −80°C until analyzed. Cytokines were quantified by a multiplex Luminex assay (University of Virginia Flow Cytometry Core Facility).

### In vivo antibody treatments

For in vivo CD86 and CD80 blockade, mice were given 200 µg anti-CD86 (clone GL-1 from Bio X Cell) or anti-CD80 (clone 16-10A1 from Bio X Cell) via i.p. injection at noted day post influenza infection. For neutrophil depletion, mice were injected i.p. with 50 µg anti-Ly6G (clone 1A8 from Bio X Cell) at the indicated days post influenza infection. Matching concentration of isotype antibody was injected for control mice.

### Virus titer

We used a tissue culture infectious dose 50 (TCID_50_) assay followed by a hemagglutination assay to quantify infectious virus in BAL fluid, as previously described [17]. In brief, we infected Madin-Darby canine kidney cells with 10-fold dilutions of BAL fluid from infected mice, then incubated the cultures for 3 days at 37°C. Supernatants were collected and mixed with a half volume of 1% chicken red blood cells (Charles River Spafas) in PBS, and TCID_50_ units were calculated from hemagglutination patterns.

### Irradiation and BM transfer

Mice were irradiated with 1050 rads and, within 24 hours, received an i.v. infusion of red blood cell-lysed bone marrow cells (1×10^6^ cells) from uninfected DEREG mice. DEREG bone marrow chimeras were allowed to reconstitute for 8 weeks before IAV infection.

### Adoptive transfer of Treg cells

CD25^+^CD4^+^ Treg cells were isolated from spleens of uninfected mice using the MACS Regulatory T cell Isolation kit (Milltenyi) according to the manufacturer's instruction. A total of 2×10^6^Tregs or CD25^−^CD4^+^ control cells were injected i.v. at indicated days post influenza infection.

### In vitro BMDC re-stimulation and intracellular cytokine staining

PR8-infected BMDCs were generated as follows: total bone marrow was isolated from uninfected mice and cultured in the presence of GM-CSF for one week to generate BMDCs. On day 7 of culture, BMDCs were harvested and incubated overnight with 1 mL PR8 virus stock at 37°C, thus loading both class I and class II MHC molecules with viral antigen. The following day, BMDCs were washed to remove free virions and subsequently cultured with lung cell suspensions in a 3∶2 ratio for 5 hours in the presence of GolgiStop (BD). After culture, cells were stained for surface markers then fixed and permeablized (BD Cytofix/Cytoperm) and stained for intracellular cytokines.

### Statistics

Unless otherwise noted, a student T test was used to compare two treatment groups. Groups larger than two were analyzed with the one-way analysis of variance test. Comparisons of two or more groups over time were analyzed with the two-way analysis of variance test followed by the Bonferroni post-test. These statistical analyses were performed using Prism5 software (for Windows; GraphPad Software, Inc.). Results are expressed as means ± SEM. Values of P<0.05 were considered statistically significant (*).

### Ethics statement

All animal experiments were conducted in accordance with the Animal Welfare Act (Public Law 91–579) and the recommendations in the Guide for the Care and Use of Laboratory Animals of the National Institutes of Health (OLAW/NIH, 2002). All animal experiments were carried out in accordance with protocols approved by the University of Virginia Animal Care and Use Committee (Protocol Number 2230).

For anesthesia, a mixture of Ketamine(20 mg/ml)/Xylazine (2 mg/ml) was injected intraperitoneally. Mice were euthanized by cervical dislocation.

## Supporting Information

Figure S1 **CD86 blockade does not affect the antigen specific T effector cell response.** Balb/c mice were infected with 0.1LD_50_ PR8 and treated with 200 µg αCD86 on day 9 p.i. Lung cell suspensions were harvested on day 12 p.i., and cells were re-stimulated with PR8 infected BMDCs in a five hour co-culture in the presence of monensin. IFNγ expression in Thy1.2^+^ CD4^+^ or CD8^+^ T cells was measured by intracellular cytokine staining (n = 2–3).
(TIF)

Figure S2 **CD86 blockade does not alter Treg function.** Balb/c mice were infected with 0.1LD_50_ PR8 and treated with 200 µg αCD86 on day 9 p.i. (A) Lung cell suspensions were harvested on day 14 p.i. and CD25 expression was analyzed on the surface of FoxP3^+^Treg cells by flow cytometry (n = 4–6, combined from 2 independent experiments). (B–D) Lung cell suspensions were harvested on day 12 p.i., and (B) cells were evaluated for FoxP3^+^Tregs, or (C) cells were re-stimulated with PR8 infected BMDCs in a five hour co-culture in the presence of monensin. IL-10 expression in FoxP3^+^ T cells was measured by intracellular cytokine staining (n = 2–3). (D) 100 µg/ml αCD86 or IgG was included added to in vitro BMDC/lung suspension co-cultures, and FoxP3^+^ T cell IL-10 expression was evaluated after a 5 hour re-stimulation (n = 5).
(TIF)

Figure S3 **CD28, CTLA4, and CD86 expression on Tregs.** Balb/c mice were infected with 0.1 LD_50_ PR8, and single cell suspensions were harvested from lung, draining lymph node, or BAL on day 10 p.i. (A) Representative histograms of surface CD28, intracellular CTLA-4, and surface CD86 expression in Tregs. (B) Percent expression of CD28, CTLA-4, and CD86 on Tregs (C) Lung cells were harvested at various days p.i., and surface CD86 expression was analyzed on FoxP3^+^CD4^+^Thy1.2^+^ T cells (A–C: n = 2, representative of 2 independent experiments).
(TIF)

Figure S4 **Treg depletion in DEREG mice.** DEREG BM chimeric mice were infected with 0.1 LD_50_ PR8 then injected with 40 ug/kg DT on day 9 p.i. (A) Lung cell suspensions from day 11, 13, and 15 were stained intracellularly for FoxP3 then evaluated by flow cytometry (n = 2–3). Representative flow plots are from day 15. (B) qRT-PCR for the influenza *PA* gene from whole lung homogenates on various days p.i. after DT treatment in DEREG mice (n = 2–4, combined from 2 independent experiments).
(TIF)

Figure S5 **CD86 expression on transferred Treg cells.** Spleens from uninfected Balb/c mice were harvested, and CD86 expression was analyzed on CD4^+^CD25^+^ T cells by flow cytometry (data is representative of 2 independent experiments).
(TIF)
