# Supplementary figures and images for: Late Engagement of CD86 after Influenza Virus Clearance Promotes Recovery in a FoxP3+ Regulatory T Cell Dependent Manner
Source: PLoS Pathog. 2014 Aug 21;10(8):e1004315. doi: 10.1371/journal.ppat.1004315 (PMC4140856; doi:10.1371/journal.ppat.1004315)

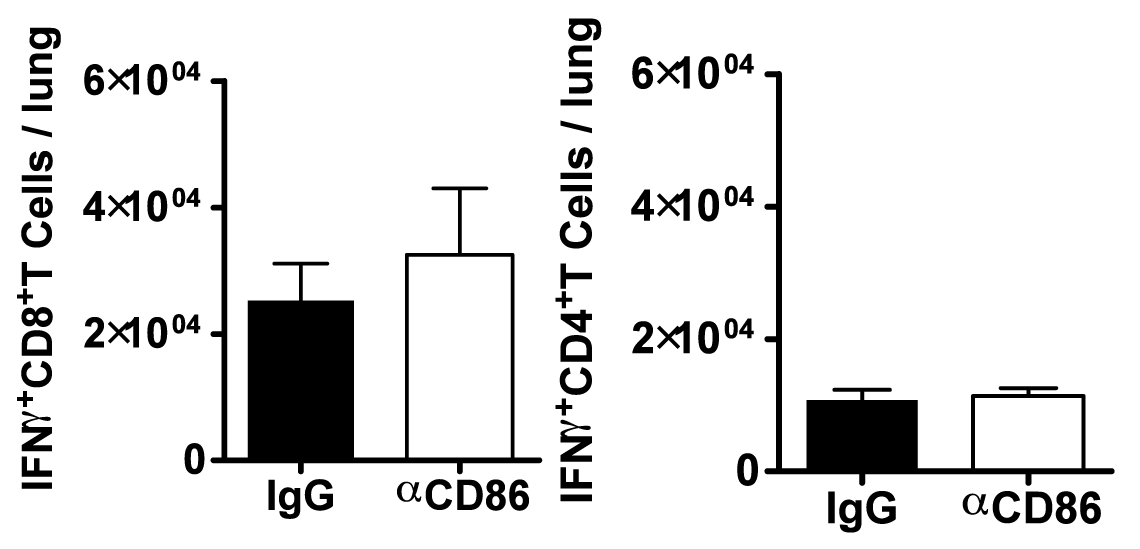

Supplement: Figure S1 — CD86 blockade does not affect the antigen specific T effector cell response. Balb/c mice were infected with 0.1LD50 PR8 and treated with 200 µg αCD86 on day 9 p.i. Lung cell suspensions were harvested on day 12 p.i., and cells were re-stimulated with PR8 infected BMDCs in a five hour co-culture in the presence of monensin. IFNγ expression in Thy1.2+ CD4+ or CD8+ T cells was measured by intracellular cytokine staining (n = 2–3). (TIF) [file ppat.1004315.s001.tif]

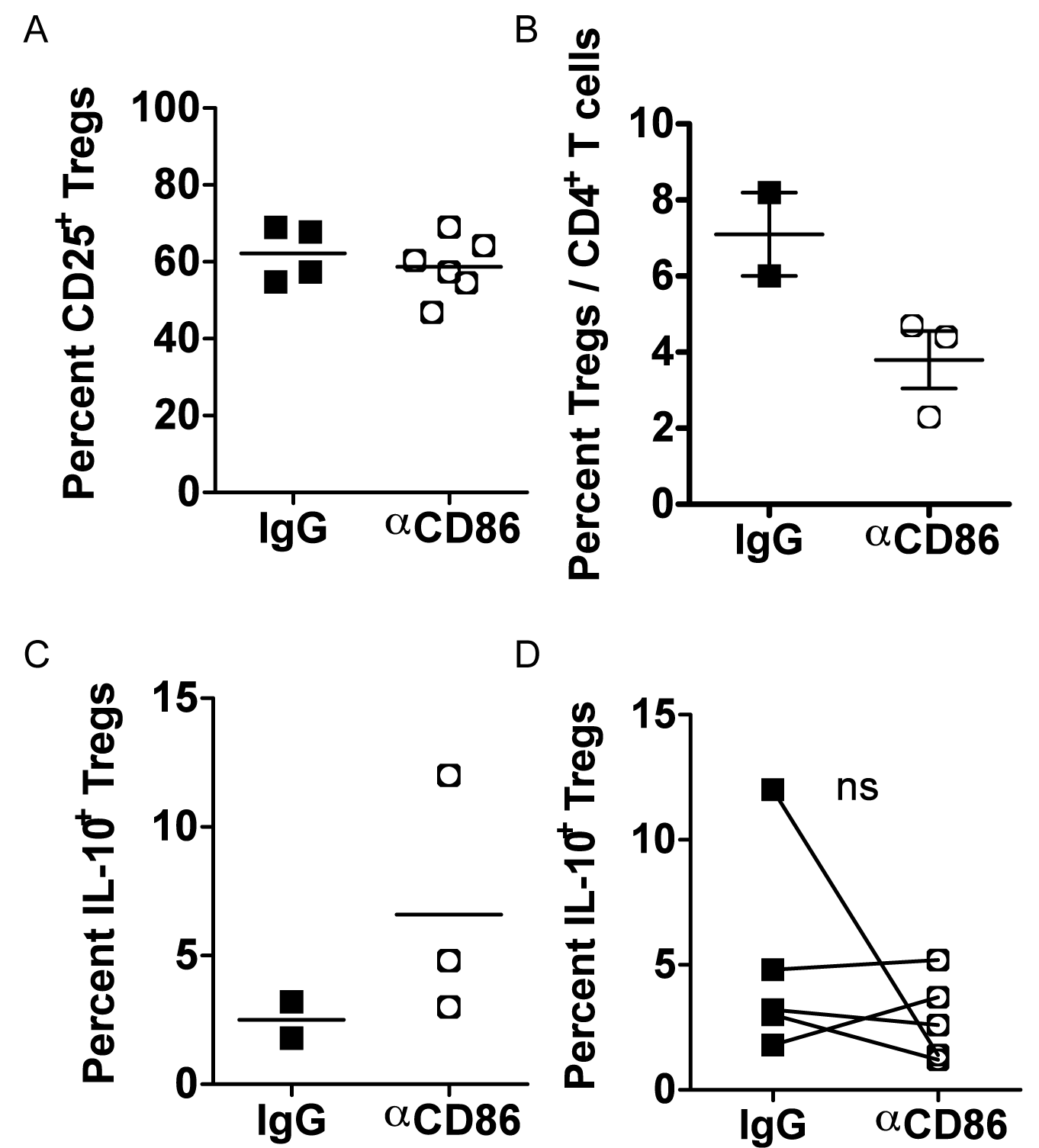

Supplement: Figure S2 — CD86 blockade does not alter Treg function. Balb/c mice were infected with 0.1LD50 PR8 and treated with 200 µg αCD86 on day 9 p.i. (A) Lung cell suspensions were harvested on day 14 p.i. and CD25 expression was analyzed on the surface of FoxP3+Treg cells by flow cytometry (n = 4–6, combined from 2 independent experiments). (B–D) Lung cell suspensions were harvested on day 12 p.i., and (B) cells were evaluated for FoxP3+Tregs, or (C) cells were re-stimulated with PR8 infected BMDCs in a five hour co-culture in the presence of monensin. IL-10 expression in FoxP3+ T cells was measured by intracellular cytokine staining (n = 2–3). (D) 100 µg/ml αCD86 or IgG was included added to in vitro BMDC/lung suspension co-cultures, and FoxP3+ T cell IL-10 expression was evaluated after a 5 hour re-stimulation (n = 5). (TIF) [file ppat.1004315.s002.tif]

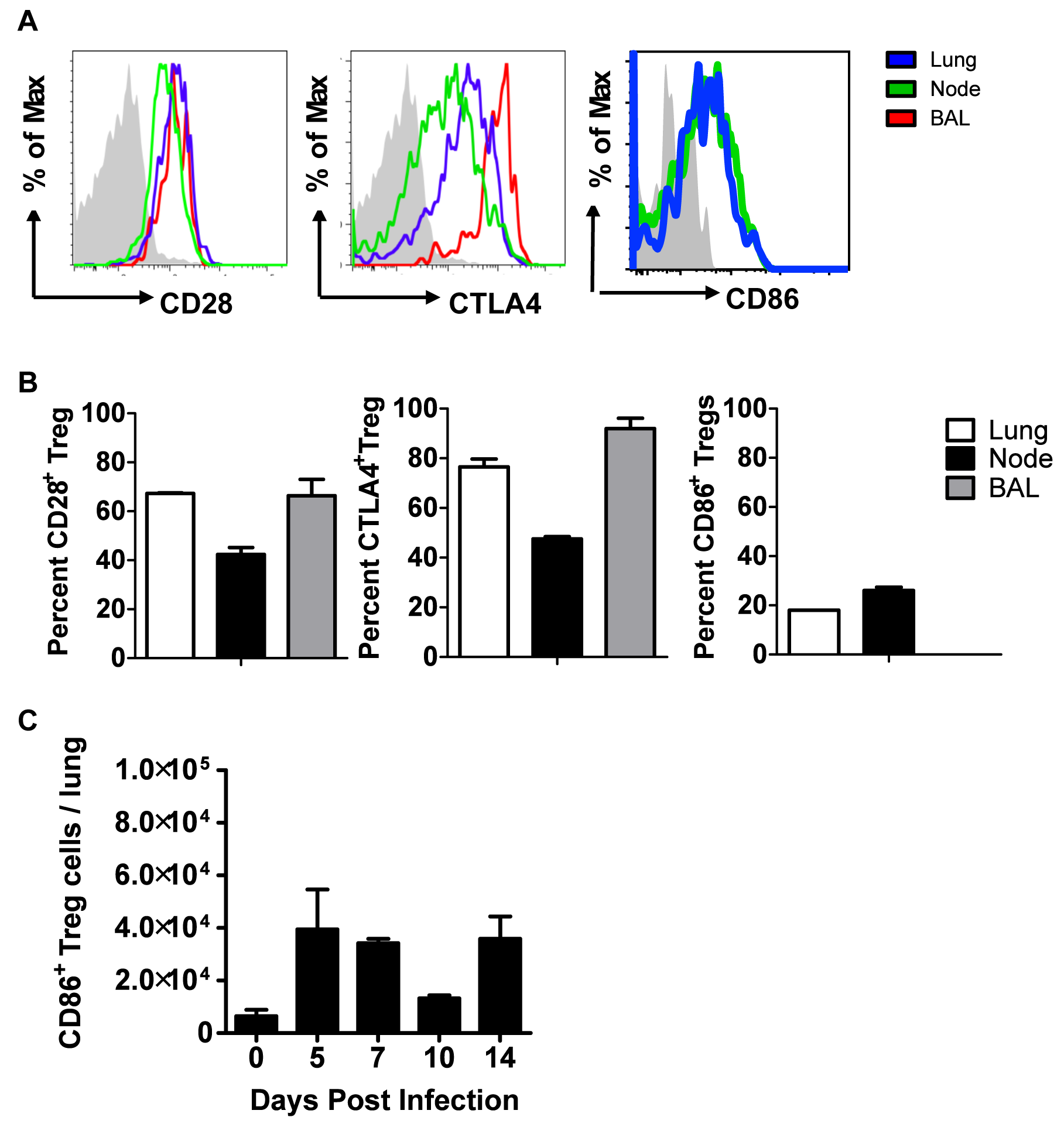

Supplement: Figure S3 — CD28, CTLA4, and CD86 expression on Tregs. Balb/c mice were infected with 0.1 LD50 PR8, and single cell suspensions were harvested from lung, draining lymph node, or BAL on day 10 p.i. (A) Representative histograms of surface CD28, intracellular CTLA-4, and surface CD86 expression in Tregs. (B) Percent expression of CD28, CTLA-4, and CD86 on Tregs (C) Lung cells were harvested at various days p.i., and surface CD86 expression was analyzed on FoxP3+CD4+Thy1.2+ T cells (A–C: n = 2, representative of 2 independent experiments). (TIF) [file ppat.1004315.s003.tif]

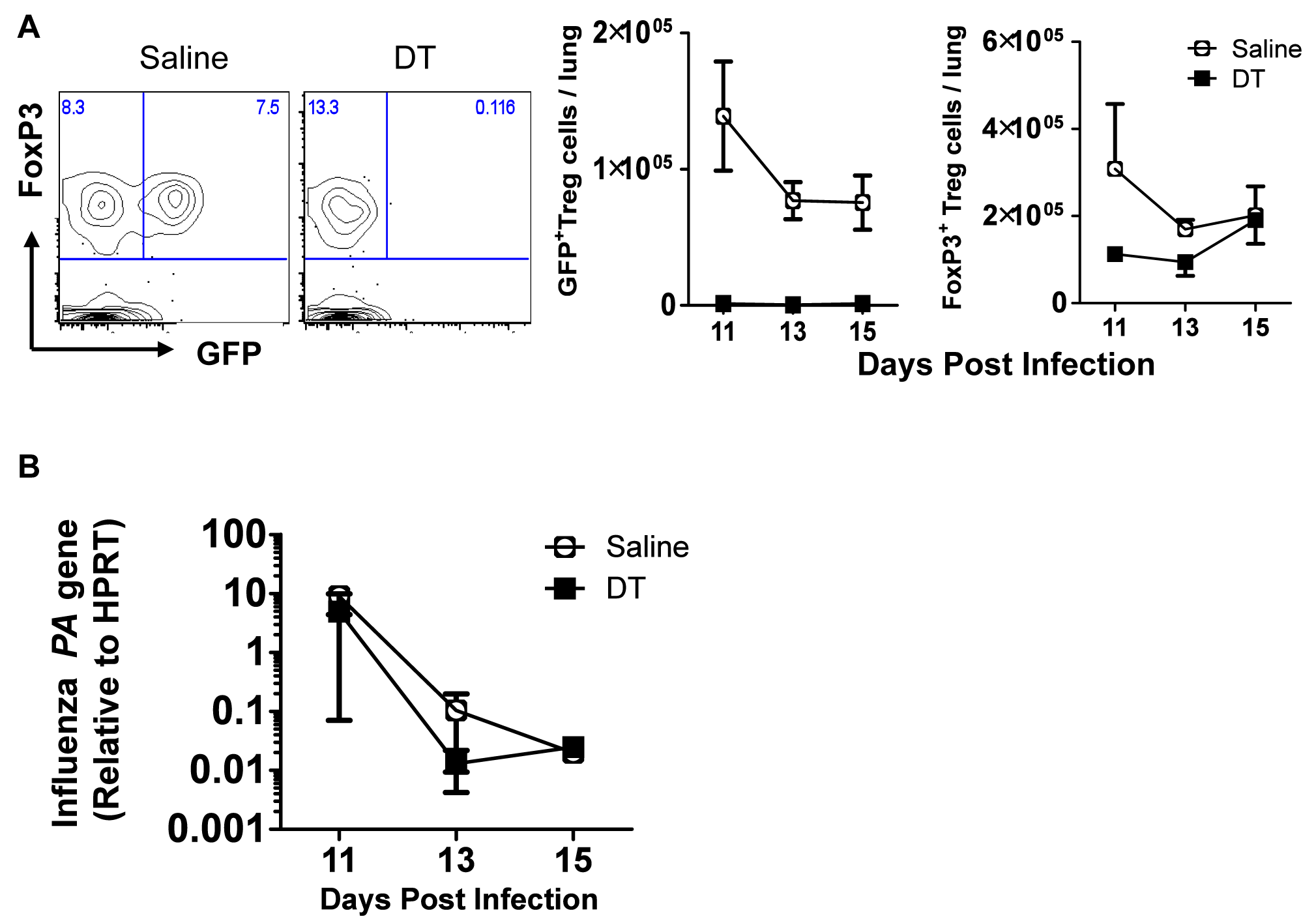

Supplement: Figure S4 — Treg depletion in DEREG mice. DEREG BM chimeric mice were infected with 0.1 LD50 PR8 then injected with 40 ug/kg DT on day 9 p.i. (A) Lung cell suspensions from day 11, 13, and 15 were stained intracellularly for FoxP3 then evaluated by flow cytometry (n = 2–3). Representative flow plots are from day 15. (B) qRT-PCR for the influenza PA gene from whole lung homogenates on various days p.i. after DT treatment in DEREG mice (n = 2–4, combined from 2 independent experiments). (TIF) [file ppat.1004315.s004.tif]

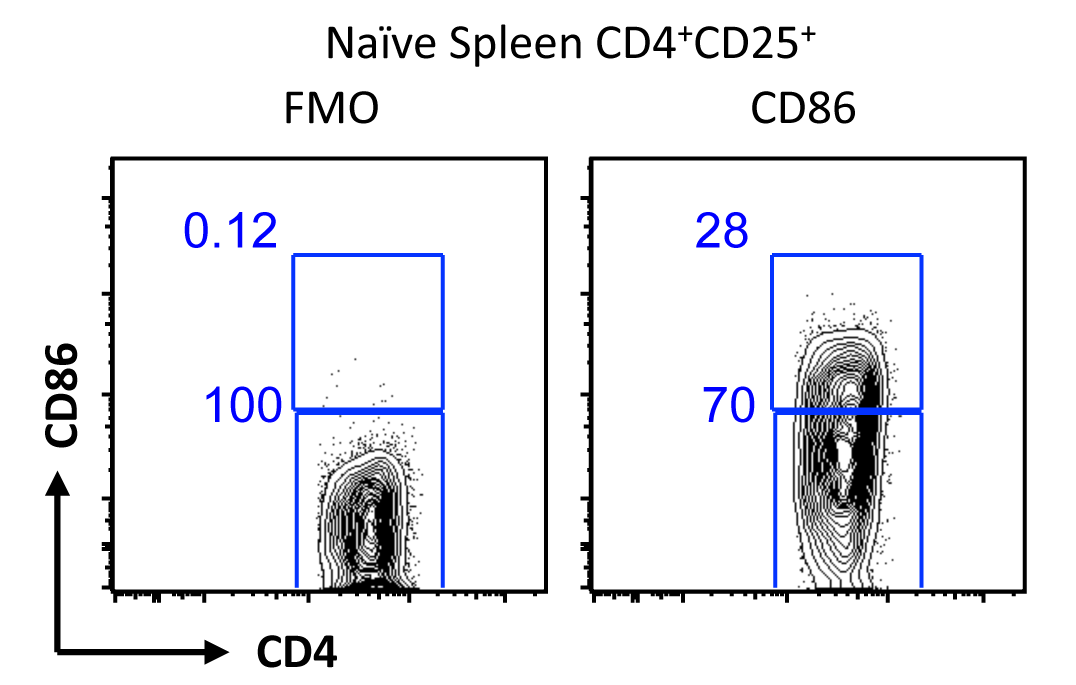

Supplement: Figure S5 — CD86 expression on transferred Treg cells. Spleens from uninfected Balb/c mice were harvested, and CD86 expression was analyzed on CD4+CD25+ T cells by flow cytometry (data is representative of 2 independent experiments). (TIF) [file ppat.1004315.s005.tif]
